# Transcriptome Reveals the Dynamic Response Mechanism of Pearl Millet Roots under Drought Stress

**DOI:** 10.3390/genes12121988

**Published:** 2021-12-15

**Authors:** Yang Ji, Xiaowen Lu, Huan Zhang, Dan Luo, Ailing Zhang, Min Sun, Qing Wu, Xiaoshan Wang, Linkai Huang

**Affiliations:** 1 Sichuan Animal Science Academy, Chengdu 610066, China; jiyang221@163.com; 2 College of Grassland Science and Technology, Sichuan Agricultural University, Chengdu 611130, China; luxiaowen1126@163.com (X.L.); zhanghuanSichuan@outlook.com (H.Z.); luodan_1111@163.com (D.L.); 18328518637@163.com (A.Z.); summin1028@163.com (M.S.); wangqiqi_shan@126.com (X.W.); 3 Department of Aquaculture, College of Animal Science and Technology, Sichuan Agricultural University, Chengdu 611130, China; byemax@163.com

**Keywords:** pearl millet, root, transcriptome, plant hormone signal transduction, ABA

## Abstract

Drought is a major threat to global agricultural production that limits the growth, development and survival rate of plants, leading to tremendous losses in yield. Pearl millet (*Cenchrus americanus* (L.) Morrone) has an excellent drought tolerance, and is an ideal plant material for studying the drought resistance of cereal crops. The roots are crucial organs of plants that experience drought stress, and the roots can sense and respond to such conditions. In this study, we explored the mechanism of drought tolerance of pearl millet by comparing transcriptomic data under normal conditions and drought treatment at four time points (24 h, 48 h, 96 h, and 144 h) in the roots during the seedling stage. A total of 1297, 2814, 7401, and 14,480 differentially expressed genes (DEGs) were found at 24 h, 48 h, 96 h, and 144 h, respectively. Based on Kyoto Encyclopedia of Genes and Genomes and Gene Ontology enrichment analyses, we found that many DEGs participated in plant hormone-related signaling pathways and the “oxidoreductase activity” pathway. These results should provide a theoretical basis to enhance drought resistance in other plant species.

## 1. Introduction

Drought is a major threat to global agricultural production that limits the growth, development and survival rate of plants, leading to tremendous losses in yield [1,2,3,4]. It can cause morphological changes and cell damage in plants [5,6], affecting the mechanisms of networks of gene expression in plants and causing many physiological and metabolic processes to be reprogrammed in response to stress [7]. Drought stress induces changes in plant metabolism: photosynthesis, the growth rate, tissue osmotic potential and antioxidant defenses [8]. To minimize the negative effects of drought stress, plants have various signaling pathways and respond by changing their growth patterns, upregulating antioxidants, accumulating osmoprotectants and producing stress proteins and chaperones [9]. In recent years, the frequent occurrence of water deficiency has had a negative impact on the production of crops [10,11]. Under drought stress at the seedling sand reproductive stages, the yield of wheat (*Triticum aestivum* L.) decreased by 40% [12,13,14]. In addition, drought also caused yield losses of 15–50% in rice (*Oryza sativa* L.), reduced the production of tea by 14–33% and could increase the mortality of tea plants by 6–19% [15,16,17,18]. Beyond that, drought can also affect the quality of crops. It was shown that drought perturbs the metabolism and growth of grapevines, potato, and tea, affective the quality of plants [19,20,21]. Above all, the increased variability in climate caused by global warming has exacerbated the frequency and severity of drought in many parts of the world in recent decades, and it is expected that drought will intensify with global warming [22,23,24,25,26]. Therefore, improving the drought resistance of crops is urgent.

Pearl millet (*Pennisetum glaucum* (L.) R. Br.) (2n = 14) is the sixth most important cereal crop in the world after rice, wheat, maize, barley and sorghum. It is grown as a staple food crop in the hottest and driest parts of sub-Saharan Africa and the Indian subcontinent [27,28]. In addition, it can also be used as a high-yield, high-quality forage crop for grazing, and it is chopped or harvested as silage for cattle and sheep in the United States, Australia, Canada and other countries [29]. Pearl millet is a promising crop for some relatively poor regions and meets the demand for a lucrative, low-risk livestock feed crop. Currently, it is cultivated over approximately 28 million hectares worldwide with grain production exceeding 22 million tons a year, and is responsible for the food and income of more than 9000 people in approximately 30 countries [30,31]. More importantly, it also has a high nutritional value, with an average of 92.5% dry matter, 2.1% ash, 2.8% crude fiber, 7.8% crude fat, 13.6% crude protein, an eight- to fifteen-fold greater α-amylase activity compared with that of wheat, and high iron levels [32]. Pearl millet is grown in areas with very limited rainfall (300–500 mm in the majority of cases) where crops, such as maize or sorghum, are not likely to survive [33]. In contrast, pearl millet appears to be the most tolerant to drought and high temperatures of most grain crops due to adaptive evolution and natural selection [33]. Therefore, pearl millet is an ideal plant material for studying the drought resistance of cereal crops.

The root system is the first organ of plants that experiences drought stress, and the roots can sense and respond to such stress conditions [34]. Currently, some progress has been made in the study of the drought resistance of pearl millet, but its complex mechanism of drought resistance makes the study of its metabolism more difficult. Therefore, research on this crop was hampered and started late; the understanding of the mechanisms for the molecular regulation of drought resistance in pearl millet roots is still limited. Therefore, the exploration of key genes of drought resistance, and the mechanisms of the molecular responses of pearl millet roots, merit further research. In this study, we analyzed genes that were differentially expressed after drought treatment at different time points (24 h, 48 h, 96 h, and 144 h) in the roots of pearl millet using RNA-Seq. These differentially expressed genes (DEGs) were then further analyzed to discover some key genes related to drought resistance. The results provided a theoretical basis for analyzing the genetic mechanism of drought resistance and guiding the breeding of drought-resistant pearl millet.

## 2. Methods

### 2.1. Plant Material Culture and Treatment

“Tifleaf 3”, provided by Beijing Mammoth Seed Company (Beijing, China), is a variety of pearl millet that was used in this study. The pearl millet seeds were sown in 20 plastic pots (10 × 15 cm) half filled with quartz sand and grown in an artificial growth chamber under 14 h of light at 26 °C and 10 h of darkness at 22 °C. Two grams (~240 seeds) were sown in each pot. In the first 3 days, the seedlings grew with just distilled water, while they were watered with 0.5× Hoagland nutrient solution after most of the seeds had germinated (the fourth day after sowing). After a 14-day growth period, during which most of the plants grew to a three-leaf period, half of the plants (10 pots) were treated with drought stress, while the other half continued to grow in normal conditions. Plants in the drought stress group were watered with a 20% polyethylene glycol 6000 (PEG) solution that was created by dissolving PEG in 0.5× Hoagland solution. The roots of pearl millet at 24 h, 48 h, 96 h, and 144 h after treatment were collected, placed in centrifuge tubes, and immediately stored at −80 °C. There were three biological replicates for each treatment, totaling 24 samples.

### 2.2. RNA Extraction and cDNA Library Construction

RNA was extracted using an RNeasy Plant Mini Kit following the manufacturer’s instructions, and its quality was checked by RNA gel electrophoresis. The purity and concentration of RNA were detected by NanoDrop spectrophotometry (Fremont, CA, USA) and a Qubit 2.0 fluorometry system, respectively. The RNA purity was measured using a NanoDrop spectrophotometer (Fremont, CA, USA), and the concentration of RNA was measured using a Qubit RNA Detection Lit in a Qubit 2.0 fluorometry system (Fremont, CA, USA). The library was constructed using an NEBNext^®^ Ultra^TM^ Directional RNA Library Prep Kit for Illumina^®^ (San Diego, CA, USA). The mRNA was enriched by an NEBNext^®^Poly (A) mRNA Magnetic Isolation Module and broken into short fragments using a fragment buffer. The first cDNA strand was synthesized using random hexamer primers, and the second strand was synthesized by adding dNTPs, DNA polymerase I, and buffer. Both cDNA strands were purified using AMPure XP beads, and the ends were repaired. The tails were added for sequencing, and the fragment size was screened using AMPure XP beads. In the end, the cDNA library was obtained by PCR enrichment. The quality control and quantification of the cDNA library were conducted on a caliper LabChip GX using an HT DNA high-sensitivity Assay Kit. RNA-Seq was performed using an Illumina HiSeq2000 platform for sequencing. A total of 24 RNA-Seq libraries were constructed in this study using pair-end sequencing technology. Each data set had an average of 1.24 × 10^7^ bp reads, 55.10% GC, and 92.91% Q20 (Supplementary Table S9).

### 2.3. Identification and Functional Analysis of Differentially Expressed Genes

The identification of level of gene expression of each sample was conducted using Kallisto software (version 0.42.3). The clean data produced by Illumina sequencing were mapped to PacBio sequencing data (SRR11816223) of pearl millet. The read count of each gene was then obtained from the mapping results, and the read count value for each gene was converted to the Transcripts per Kilobase Million (TPM value). DEGs were screened by *p* < 0.05 and |log_2_ (FC)| ≥ 1. The DEGs were subjected to enrichment analysis using Gene Ontology (GO) and Kyoto Encyclopedia of Genes and Genomes (KEGG). All the DEGs were analyzed using the R (V3.3.0) weighted gene co-expression network analysis (WGCNA) package.

## 3. Results

### 3.1. Identification of Differentially Expressed Genes 

**Figure 1:**
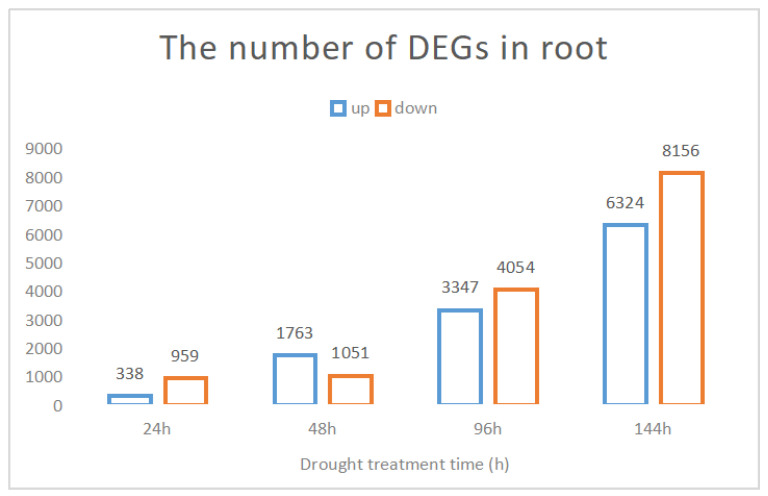
The number of DEGs under four time points in the roots of pearl millet after drought stress. The blue bars represent the number of upregulated genes, and the orange bars represent the number of downregulated genes. DEGs, differentially expressed genes.

To elucidate the genes that responded to drought stress in the root and the dynamic molecular mechanisms of drought resistance of pearl millet, we identified the DEGs that were differentially expressed under drought treatment at 24 h, 48 h, 96 h, and 144 h. The results showed that the number of DEGs at 24 h, 48 h, 96 h, and 144 h was 1297, 2814, 7401, and 14,480, respectively. The number of upregulated genes was 338, 1763, 3347 and 6324, respectively, and the number of downregulated genes was 959, 1051, 4054 and 8156, respectively (Figure 1). 

**Figure 2:**
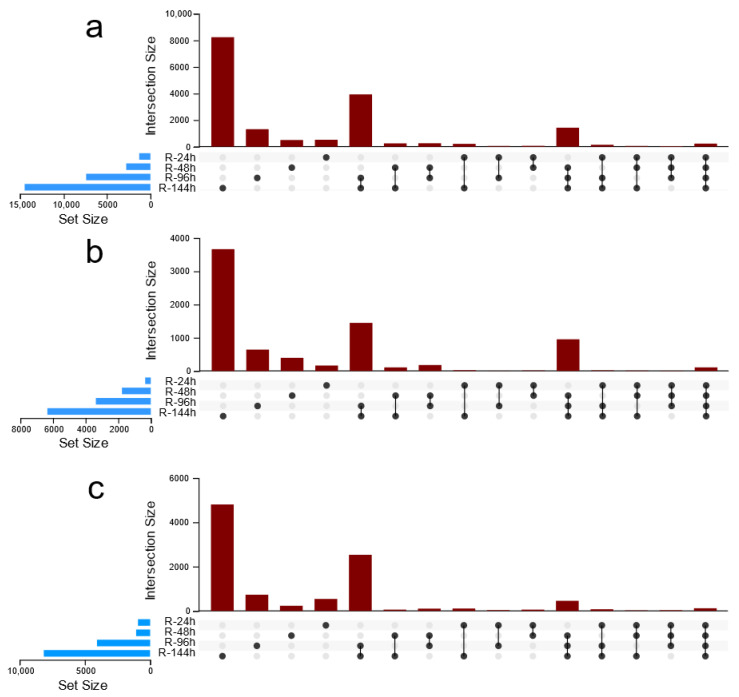
Venn diagram of differentially expressed genes at each point. (**a**) All DEGs; (**b**) Upregulated DEGs; (**c**) Downregulated DEGs; R: root. DEGs, differentially expressed genes.

A Venn diagram analysis was performed on the total DEGs and the upregulated and downregulated genes at four time points (Figure 2). A total of 229 genes were found to be differentially expressed from 24 h to 144 h. There were 115 genes that were downregulated from 24 h to 144 h, while 105 genes were upregulated from 24 h to 144 h. Five genes (*i0_HQ_LWC_c217/f2p0/661*, *i0_LQ_LWC_c1867/f1p0/760*, *i0_LQ_LWC_c751/f1p0/776* (responsive to abscisic acid (RAB), *i0_LQ_LWC_c982/f1p0/940* and *i2_LQ_LWC_c50304/f1p2/2779*) was more than a ten-fold upregulation at 144 h. 

### 3.2. KEGG Enrichment Analysis of DEGs

**Figure 3:**
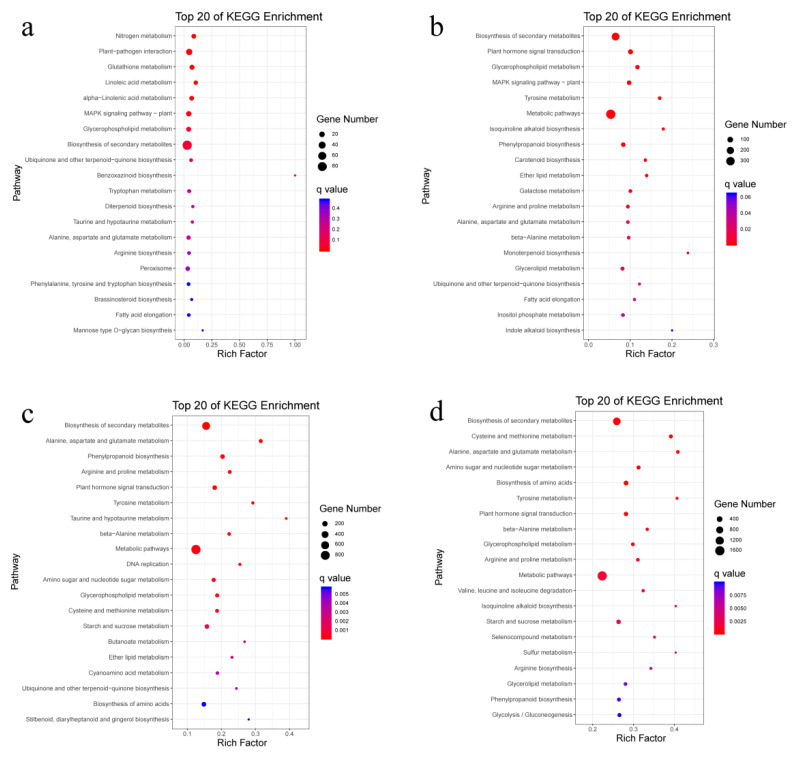
KEGG enrichment analysis of DEGs at each time point in the roots. (**a**–**d**) represent gene enrichments at 24 h, 48 h, 96 h and 144 h, respectively. DEGs, differentially expressed genes; KEGG, Kyoto Encyclopedia of Genes and Genomes.

We performed a KEGG enrichment analysis of the DEGs in roots of pearl millet at four time points to gain an insight into their potential functions (Figure 3). It is worth noting that the drought-induced DEGs were significantly enriched in plant hormone-related signaling pathways at 48 h, 96 h, and 144 h. In addition to the pathways described above, these DEGs were significantly enriched in six pathways, including “Nitrogen metabolism”, “Plant pathogen interaction”, “Glutathione metabolism”, “Linoleic acid metabolism” and “Alpha-Linolenic acid metabolism” at 24 h (Figure 3a). As the drought time extended, the number of DEGs increased. At 48 h (Figure 3b), the DEGs were significantly enriched in 19 pathways, including “Biosynthesis of secondary metabolites”, “Glycerophospholipid metabolism”, and “Tyrosine metabolism”, among others. At 96 h (Figure 3c), the DEGs were significantly enriched in 28 pathways, including "Biosynthesis of secondary metabolites”, “Metabolism of alanine, aspartic acid, and glutamic acid”, and “Biosynthesis of phenylpropane”, among others. There was a total of 34 pathways in which DEGs were significantly enriched when the drought time was prolonged to 144 h, including “Biosynthesis of secondary metabolites”, “Cysteine and methionine metabolism” and “Alanine, aspartic acid, and glutamic acid metabolism”, among others (Figure 3d).

### 3.3. Analysis of ABA Hormone Transduction and Zeatin Signal Transduction Related Genes

**Figure 4:**
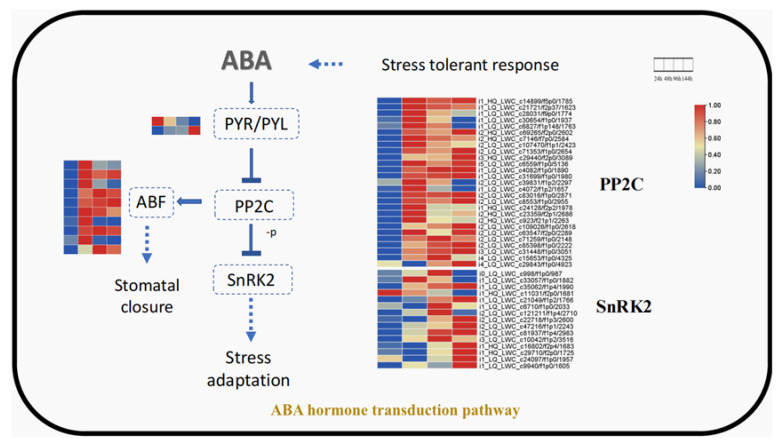
Diagram of the ABA signal transduction pathway of pearl millet roots. The small grid in each column in the figure represents the log2(FC) value of the gene, and the log2(FC) of each row is Zero to One. -p, dephosphorylation; ABA, abscisic acid; FC, fold-change; PYR/PYL, pyrabatin riesistance/pyrabatin riesistance 1-like.

The enrichment analysis of the pearl millet root signaling pathway that revealed significant enrichment of genes involved in the “plant hormone signaling pathway” and “ABA hormone transduction” were analyzed in more detail (Figure 4). In this experiment, we found that two ABA receptor *PYL* genes (*i2_LQ_LWC_c3719/f1p0/2678* and *i1_HQ_LWC_c37349/f3p10/1291*) were differentially expressed in the roots under drought stress. *i2_LQ_LWC_c3719/f1p0/2678* was downregulated at 48 h, 96 h and 144 h, while *i1_HQ_LWC_c37349/f3p10/1291* was upregulated at 144 h. A total of 27 *PP2C* genes were differentially expressed in the roots. Most were upregulated from 48 h to 144 h, and 10 *PP2C* genes saw more than a five-fold upregulation. There were 15 *SnRK2* genes that were differentially expressed at 96 h and 144 h in the root, and 14 of the 15 genes were upregulated.

CRE1, AHP, ARR-A, and ARR-B are the signaling molecules of zeatin signal transduction. In this study, we found that four *CRE1* genes were downregulated, with *i1_HQ_LWC_c3032/f1p5/4571* seeing a 19-fold downregulation after 144 h of drought treatment. Five *ARR-A* genes were downregulated and nine *ARR-B* genes were downregulated. In general, most of the DEGs in the zeatin signaling pathway were downregulated under drought stress.

### 3.4. GO Enrichment Analysis of the DEGs 

**Figure 5:**
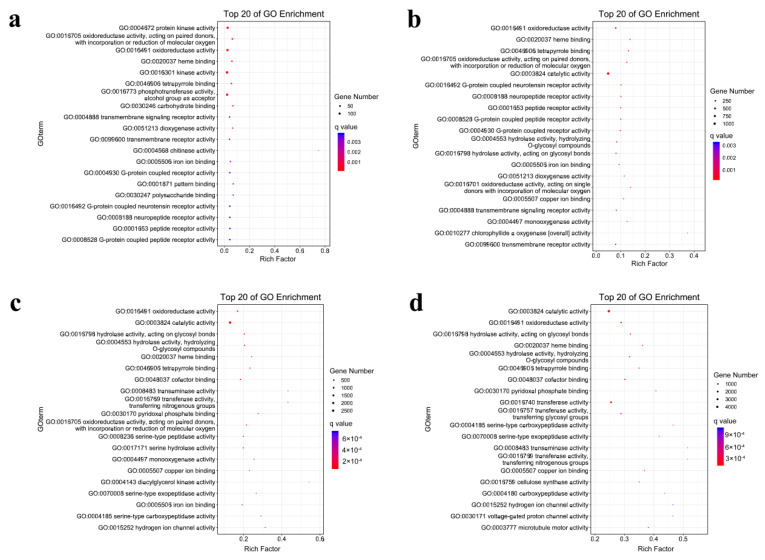
GO enrichment analysis of the DEGs in roots: (**a**–**d**) represent gene enrichments at 24 h, 48 h, 96 h and 144 h, respectively. DEGS, differentially expressed genes; GO, gene ontology.

A GO enrichment analysis was also performed on DEGs at each time point in the roots to further elucidate their function (Figure 5). When the drought stress lasted for 24 h, the DEGs were significantly enriched into 41 pathways, including “protein kinase activity”, “oxidoreductase activity”, and “heme binding”, among others. When the drought stress duration was extended to 48 h, the GO pathways were significantly enriched with the increase in the number of DEGs to 46, including “oxidoreductase activity”, “heme binding”, and “tetrapyrrole binding”, among others. The pathways for the significant enrichment of DEGs in the pearl millet roots were 65 and 35, respectively, at 96 h and 144 h of drought stress (Figure 5a,b). The DEGs of 96 h enriched in “oxidoreductase activity”, “catalytic activity” and “hydrolase activity, acting on glycosyl bonds” among others. At 144 h, the pathways for the significant enrichment of DEGs were “catalytic activity”, “oxidoreductase activity” and “hydrolase activity, acting on glycosyl bonds”, among others.

## 4. Discussion

Since pearl millet is an important and drought-tolerant crop, the study of its mechanism of drought tolerance is highly important for drought-resistant breeding and research on plants. This study analyzed the DEGs of its roots under different times of drought stress. When faced with drought treatment, the number of DEGs in pearl millet roots increased as the time of stress was extended. This may be due to an increase in the degree of drought stress on plants as the time was extended. Therefore, the plant needs to synthesize certain compounds through the differential expression of more genes to improve its response to drought stress. In the Venn analysis, we found that five genes experienced more than a 10-fold upregulation at all the time points. These genes may play essential roles in the drought response of pearl millet. In addition, one of these genes (*i0_LQ_LWC_c751/f1p0/776*) was annotated as an *RAB* (responsive to ABA) gene. A substantial amount of research shows that RAB proteins are related to environmental stresses, such as cold, salt and drought [35,36,37,38,39]. Thus, we can reasonably hypothesize that this *RAB* gene may play a key role in the drought tolerance of pearl millet. However, its function merits further verification. 

The KEGG enrichment analysis of DEGs in the roots at each time point showed that the DEGs were significantly enriched in the “plant hormone signal transduction pathway”. Among them, many studies found that the ABA-mediated signaling pathway is closely related to the response to drought stress [40,41,42]. ABA is produced in roots under drought stress and transported to the leaves where it plays a key role in regulating water uptake in the plant [43]. As one of the ABA signal receptors, PYL plays an important role in ABA signal transduction. After binding to ABA, it can interact with class A PP2C and inhibit PP2C phosphatase activity, thus initiating ABA signal transduction [44]. PP2C also negatively regulates SnRK2, and these three core components can also form a double negative regulatory system in the pathway of ABA signal transduction. At 96 h and 144 h, the expressions of SnRK2, such as *i1_LQ_LWC_c35062/f1p4/1990*, *i2_LQ_LWC_c47216/f1p1/2243 i1_HQ_LWC_c29710/f2p0/1725* and *i1_LQ_LWC_c21049/f1p2/1766*, produced a large number of upregulated transcripts, which could be due to the production of proteins and the transmission of signals for a certain period of time. After 144 h, the expression of PP2C was inhibited. Therefore, SnRK2 began to increase its expression, delivering the signal in response to drought. In addition, many studies show that the expression of some genes, particularly transcription factors, may increase the sensitivity of plants to ABA and increase the drought tolerance of plants. For example, a *bZIP* gene was found to be a positive regulator in the process of the response to ABA and could enhance the drought tolerance of rice [40]. The overexpression of a transcription factor, *MYB52*, in Arabidopsis was proven to increase the hypersensitivity of ABA and drought tolerance [45]. In our results, when many *PP2C* genes were upregulated, numerous *SnRK2* genes were still upregulated. Therefore, we hypothesized whether this was due to some important transcription factors in pearl millet that could regulate signal molecules, such as SnRK2, in the ABA signaling pathway to respond to drought conditions. In addition, previous studies found that ABA could have antagonistic effects on the synthesis of zein [46]. In this experiment, most DEGS in the zeatin synthesis pathway were significantly downregulated, including CRE1 (*i4_LQ_LWC_c3032/f1p5/4571*), ARR-A (*i2_LQ_LWC_c112132/f1p0/2100* *and i1_LQ_LWC_c36348/f1p0/1452*) and ARR-B (*i2_HQ_LWC_c72282/f2p2/2653*, *i2_LQ_LWC_c9650/f1p4/2652* and *i2_LQ_LWC_c34899/f1p4/2618*), which may cause an increase in the content of ABA, thus improving the drought resistance of plants at a certain time point. Our results indicated that the ABA-mediated signaling pathway played an important role in the drought resistance of pearl millet, which could also be related to the reduction in the synthesis of zein.

In the GO function enrichment analysis, we found that the DEGs were enriched into an “oxidoreductase activity” pathway, which was significant at every time point. This indicated that, compared with normal conditions, more redox reactions happened when the plants were deficient in water. Many studies showed that reactive oxygen species (ROS) accumulate in plants when they are under drought stress. Excessive ROS are chemically active and easily attack cell membranes and cell macromolecules, causing membrane peroxidation and damage to DNA and other macromolecules [47,48,49]. Therefore, plants must produce antioxidants to detoxify these ROS. The detoxification of ROS is primarily mediated by catalase, ascorbate peroxidase (APX), peroxidase (POD) and superoxide dismutase [50,51,52,53]. Our research shows that POD and APX were found to be the main differentially expressed genes in the “oxidoreductase activity” pathway, and POD was differentially expressed at every time point, with the largest number found at 96 h and 144 h. Notably, since *i1_LQ_LWC_c14033/f1p0/1417* was differentially expressed at each time point, including 24 h, 48 h, 96 h, and 144 h. In pearl millet, the production of some or all of these enzymes may be the reason that it is so effective in resisting drought.

## 5. Conclusions

In general, the mechanism of drought resistance in pearl millet is highly complex and involves a great deal of gene expression regulation. However, a substantial amount of experimental data proves that the ABA-mediated signaling pathway is significant for the drought resistance of pearl millet. Moreover, the antioxidant system, and particularly the POD, may also play an indispensable role in the drought resistance of pearl millet. Studies on the mechanisms of the drought resistance of pearl millet show that the interaction between genes is dependent on a large amount of biological information, which is used to predict the data that can provide some insights into the complex mechanisms of the drought resistance of pearl millet. However, more validation tests are necessary to determine the relationships among these many genes in more detail and to identify the exact roles that they play in the drought resistance of pearl millet.

## Data Availability

The datasets supporting the conclusions of this article are included within the article (and its supplemental files). RNA-seq database for pearl millet can be downloaded from NCBI under the accession number PRJNA766308 (https://www.ncbi.nlm.nih.gov/sra/PRJNA766308, accessed on 1 December 2021), and the data can be shared on reasonable request of the corresponding author.

## Supplementary Materials

The following are available online at https://www.mdpi.com/article/10.3390/genes12121988/s1, Tables S1–S4: GO enrichment analysis at 24 h, 48 h, 96 h and 144 h, Tables S5–S8: KEGG enrichment analysis at 24 h, 48 h, 96 h and 144 h, Table S9: Overview of the sequencing results of 24 samples.
